# Vitamin D-inducible antimicrobial peptide LL-37 binds SARS-CoV-2 Spike and accessory proteins ORF7a and ORF8

**DOI:** 10.3389/fcimb.2025.1671738

**Published:** 2025-09-23

**Authors:** Annika Roth, Steffen Lütke, Matthias Mörgelin, Denise Meinberger, Gabriele Hermes, Gerhard Sengle, Manuel Koch, Marco Drexelius, Jan Gebauer, Ines Neundorf, Dzemal Elezagic, Mats Paulsson, Thomas Streichert, Andreas R. Klatt

**Affiliations:** ^1^ Institute for Clinical Chemistry, Medical Faculty and University Hospital Cologne, University of Cologne, Cologne, Germany; ^2^ Department of Pediatrics and Adolescent Medicine, Faculty of Medicine and University Hospital Cologne, University of Cologne, Cologne, Germany; ^3^ Center for Biochemistry, Medical Faculty and University Hospital Cologne, Cologne, Germany; ^4^ Division of Infection Medicine, Department of Clinical Sciences, Lund University, Lund, Sweden; ^5^ Colzyx AB, Lund, Sweden; ^6^ Center for Molecular Medicine Cologne (CMMC), University of Cologne, Cologne, Germany; ^7^ Cologne Center for Musculoskeletal Biomechanics (CCMB), Cologne, Germany; ^8^ Cologne Excellence Cluster on Cellular Stress Responses in Ageing-Associated Diseases (CECAD), University of Cologne, Cologne, Germany; ^9^ Institute for Dental Research and Oral Musculoskeletal Biology, Faculty of Medicine and University Hospital Cologne, University of Cologne, Cologne, Germany; ^10^ Institute for Biochemistry, Department of Chemistry, Faculty of Mathematics and Natural Sciences, University of Cologne, Cologne, Germany

**Keywords:** SARS-CoV-2, COVID-19, Spike, ORF7a, ORF8, LL-37, surface plasmon resonance, vitamin D

## Abstract

**Background:**

The role of vitamin D in Coronavirus Disease 2019 (COVID-19) outcomes remains debated, but emerging evidence suggests it may enhance recovery by strengthening immune responses. Vitamin D upregulates LL-37, an antimicrobial peptide with broad antiviral activity, including potential benefits against SARS-CoV-2. LL-37’s interactions with viral proteins, however, remain incompletely understood.

**Methods:**

We investigated LL-37’s interactions with the SARS-CoV-2 Spike glycoprotein and the accessory proteins ORF7a and ORF8 using surface plasmon resonance and negative-stain electron microscopy. These approaches were employed to assess LL-37’s binding capabilities and potential impact on viral infectivity.

**Results:**

LL-37 bound multiple domains of the Spike protein and inhibited its interaction with the human angiotensin-converting enzyme 2 (hACE2) receptor *in vitro*. Up to seven LL-37 molecules were observed surrounding Spike, forming a halo-like structure that may block receptor engagement. LL-37 also bound to ORF7a and ORF8, potentially impairing their ability to disrupt host cell processes. Notably, LL-37’s interaction with ORF7a may prevent degradation of SNAP29, restoring autophagy and promoting viral clearance.

**Conclusions:**

LL-37 disrupts key viral-host interactions by binding to Spike, ORF7a, and ORF8, thereby reducing SARS-CoV-2 infectivity. These findings highlight LL-37’s potential as a therapeutic agent in COVID-19 and provide mechanistic insight into its antiviral actions.

## Introduction

Coronavirus disease 2019 (COVID-19), triggered by severe acute respiratory syndrome coronavirus type 2 (SARS-CoV-2), has by now caused more than 7 million deaths worldwide (https://covid19.who.int/). Although the lethality of COVID-19 has decreased significantly with the current Omicron variant, people still die from the disease every day (Iuliano et al., 2022). Different COVID-19 vaccines are available, which mostly target the Spike glycoprotein (Spike) or its receptor binding domain (RBD) (Dai and Gao, 2021; Mellet and Pepper, 2021). However, newly emerging SARS-CoV-2 variants are a matter of concern, since variants such as Delta and Omicron harbor mutations in Spike that affect the protective efficacy of vaccines (Accorsi et al., 2022; Chen et al., 2022). SARS-CoV-2 houses a genome of approximately 30 kb that encodes, among others, Spike. Furthermore, the genome codes for a total of six accessory proteins including open reading frame 8 (ORF8), which dimerizes, and is linked to efficient pathogen transmission (Wang et al., 2020; Hassan et al., 2021; Matsuoka et al., 2022). Besides Spike and RBD, ORF8 is a candidate antigen target for vaccine development, though displaying only a low immunogenicity (Can et al., 2020; Tang et al., 2022). Contrasting findings have been reported, however, showing that ORF8 is one of the main accessory proteins eliciting antibody responses (Hachim et al., 2020). ORF8 induces endoplasmic reticulum stress-like responses that facilitate viral replication (Wang et al., 2023). It also interferes with host immunity by downregulating MHC class I molecules, allowing the virus to evade cytotoxic T cell recognition (Vinjamuri et al., 2022). Through these combined effects, ORF8 contributes to viral persistence and pathogenesis, making it a promising target for therapeutic intervention. In addition to ORF8, the virus expresses ORF7a, a protein that enhances virulence by manipulating autophagy processes within the host cell. ORF7a initiates autophagy but simultaneously inhibits the fusion of autophagosomes with lysosomes. This disruption allows SARS-CoV-2 to evade degradation and boosts its replication, thereby hijacking the host’s cellular machinery and promoting its pathogenicity. These dual roles of ORF7a in modulating autophagy enhance viral survival and replication, contributing to the virus’s overall virulence (Hou et al., 2023). While Spike and RBD remain the primary targets of vaccine development, accessory proteins like ORF8 and ORF7a could also serve as potential antigens.

The beneficial effect of vitamin D on the infection rate and the outcome of COVID-19 is subject of controversial debate since the beginning of the pandemic. Studies suggest that treatment with vitamin D can improve the outcome and reduce intensive care unit treatment (Entrenas Castillo et al., 2020; Alcala-Diaz et al., 2021; Nogues et al., 2021). In addition, its antiviral activity has been demonstrated *in vitro* and *in vivo* against several other viruses, underscoring its potential broad therapeutic relevance in viral infections (Jaratsittisin et al., 2023; Luo et al., 2024). The effects of vitamin D are mediated by its binding to the vitamin D receptor and subsequent binding of this receptor to vitamin D response elements (Miller and Gallo, 2010). A consensus vitamin D response element is located in the promotor of the human cathelicidin gene. Therefore, cathelicidin, the precursor of the well-investigated human antimicrobial peptide LL-37, is strongly up-regulated by vitamin D (Wang et al., 2004). LL-37 consists of 37 amino acids with an overall positive net charge (+6) and can thus eliminate microbes directly by electrostatic binding to negatively charged molecules on microbial membranes. In addition, the peptide has antiviral effects including inhibition of herpes simplex virus type one replication, vaccinia virus replication, retroviral replication, and replication of some adenovirus serotypes (Pahar et al., 2020). LL-37 can prevent severe COVID-19 by directly inhibiting the virus, by promoting neutrophil extracellular trap (NET) clearance, enhancing endothelial repair as well as by modulating inflammatory responses (Aloul et al., 2022). Interaction with the ACE2 receptor, through which viral entry is mediated, has likewise been demonstrated, further emphasizing the broad antiviral versatility of LL-37 against SARS-CoV-2 (Li et al., 2021). Moreover, orally administered LL-37 shortens nucleic acid clearance time of COVID-19 patients leading to a better therapeutic outcome (Zhao et al., 2023). Given the multifaceted antiviral properties of LL-37, it is crucial to further investigate how this peptide interacts with specific viral proteins to better understand its full therapeutic potential. Binding of LL-37 to Spike was already shown (Wang et al., 2021). We hypothesize that LL-37 might interact not only with Spike but also ORF7a and ORF8, to block viral entry and prevent immune evasion more efficiently than by blocking Spike alone.

In this study, we investigated the interaction between LL-37 and Spike, different subregions of Spike (S1 and S2 subunit, RBD), ORF7a and ORF8 by surface plasmon resonance (SPR), and negative stain electron microscopy (EM). Furthermore, we examined whether LL-37 can block binding of Spike to its cellular receptor human angiotensin converting enzyme 2 (hACE2) *in vitro*.

## Material and methods

### Cloning and purification of SARS-CoV-2 proteins

**Figure 1:**
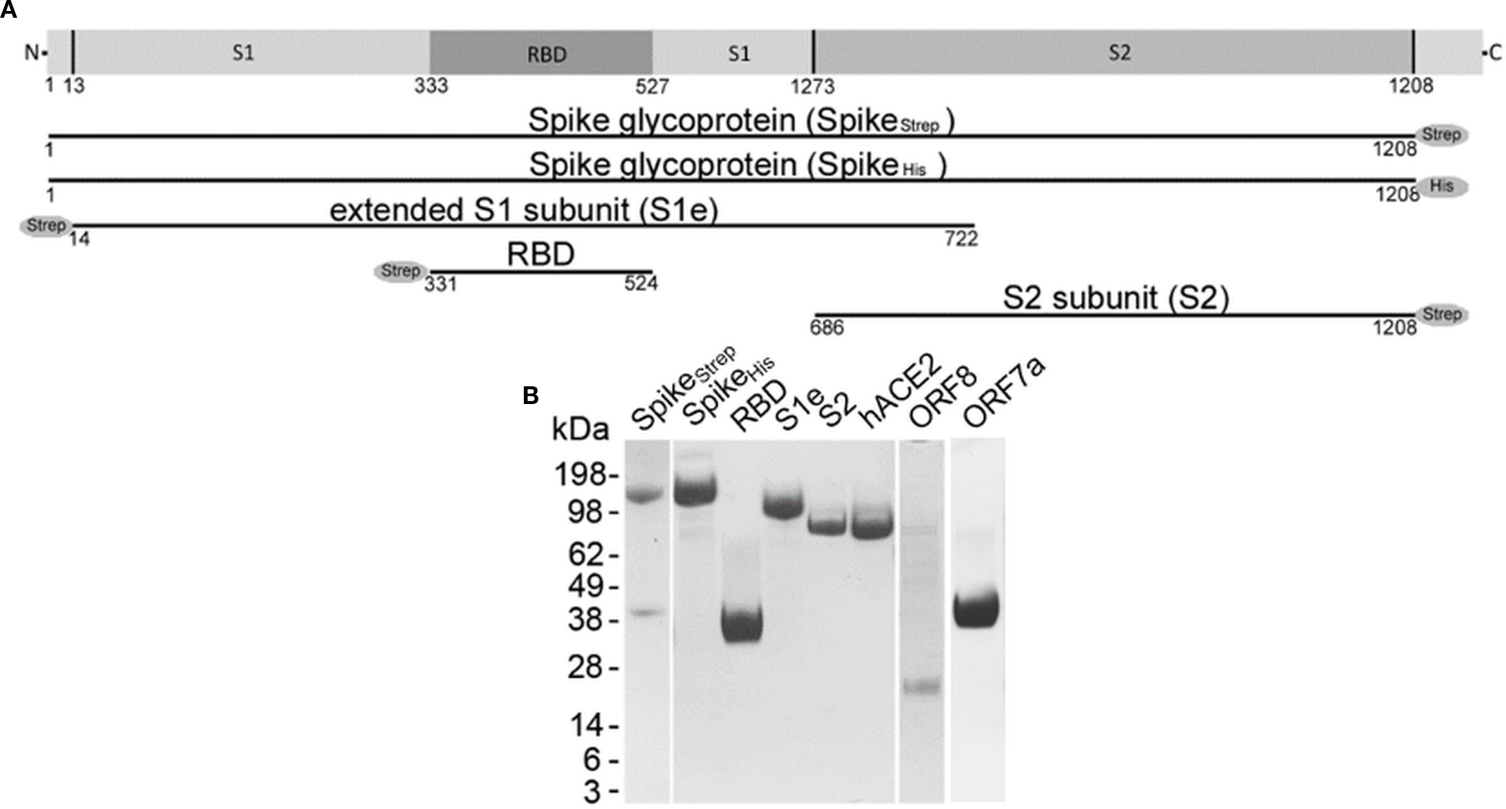
Recombinant SARS-CoV-2 proteins. **(A)** Schematic representation of recombinantly expressed Spike glycoprotein (Spike_Strep_, Spike_His_), extended S1 subunit (S1e), receptor-binding domain (RBD), and S2 subunit (S2). **(B)** A 4-12% Bis-Tris gel was loaded with 5 µg Spike_Strep_, Spike_His_, RBD, S1e, S2, human angiotensin converting enzyme 2 (hACE2), and open reading frame 8 and 7a (ORF8 and ORF7a) stained with Coomassie Brilliant Blue R 250. Pictures of gels were only cut in width and lanes are shown in their full length. All cropped lanes of different gels are divided by white spaces.

Cloning and purification of SARS-CoV-2 proteins of the Wuhan variant were performed as described previously (Meinberger et al., 2021). Additionally, genes encoding the S2 subunit (S2; MN908947; AA: 686-1207; furin site mutated K986P and V987P) including a C-terminal T4 foldon (AA: GSGYIPEAPRDGQAYVRKDGEWVLLSTFLRSL) (Wrapp et al., 2020), the extended S1 subunit (S1e; MN908947; AA: 14-722; furin site mutated K986P and V987P) (Walls et al., 2020), ORF7a (MN908947; AA: 27394-27681) (Zhou et al., 2021) and hACE2 (NM_001371415.1; AA: 20-611) (Xiao et al., 2021) were amplified from synthetic gene plasmids (GeneArt Gene Synthesis by Thermo Fisher Scientific, USA) using specific PCR-primers (**Supplementary Table S1**). After digestion with appropriate restriction enzymes (**Supplementary Table S1**) the PCR products were cloned into modified sleeping beauty transposon expression vectors (Kowarz et al., 2015) that contain a twin strep tag or a polyhistidine tag (**Figure 1a**) and a thrombin cleavage site at the C-terminus. In the case of RBD, S1e, ORF7a, ORF8, and hACE2 the tag was added at the 5’ end including a BM40 signal peptide sequence. Gene-optimized DNA sequences from the different constructs can be obtained upon request (Manuel.Koch@uni-koeln.de). The sleeping beauty transposon system was stably transfected into HEK293 EBNA cells using FuGENE HD (Promega GmbH, USA) in DMEM/F12 (Merck, Germany) supplemented with 6% FBS (Biochrom AG, Germany). Cells were quickly selected with puromycin dihydrochloride (3 µg/ml; Sigma, USA) for four days and seeded into triple flasks. After four days of induction with doxycycline hyclate (0.5 µg/ml; Sigma, USA) supernatants were harvested every third day and filtered. Recombinant proteins with a twin strep tag were purified using Strep-Tactin XT (IBA Lifescience, Germany), and eluted with biotin elution buffer (IBA Lifescience, Germany). In the case of polyhistidine-tagged proteins, cell supernatants were purified via an Indigo-Ni column (Cube Biotech, Germany), washed stepwise with imidazole, and eluted with 200 mM imidazole. Proteins were dialyzed against Tris-buffered saline or phosphate-buffered saline and aliquots were stored at 4°C.

### LL-37 and control peptide

Synthetic LL-37 (AA: LLGDFFRKSKEKIGKEFKRIVQRIKDFLRNLVPRTES) was purchased from Invivogen (USA). A synthetic control peptide with an alpha-helical structure and a length of 29 amino acids (DKDGDLKTQIEKLWTEVNALKEIQALQTVCL) was purchased from Biomatik. Lyophilized peptides were reconstituted with endotoxin-free water to reach a final concentration of 1 mg/ml. Purity was ≥ 95%.

### SDS-PAGE and Coomassie staining

Protein samples were reduced with dithiothreitol, mixed with LDS Sample Buffer (Thermo Fischer Scientific, USA), and heated to 90°C for 10 min. SDS-PAGE was performed in a 4-12% Bis-Tris polyacrylamide gel (Thermo Fischer Scientific, USA). Proteins were visualized using Coomassie Brilliant Blue R 250 (Merck, Germany).

### Interaction studies using surface plasmon resonance

SPR experiments were performed as described previously (Sengle et al., 2008a, 2008b) using a BIAcore 2000 system (BIAcore AB, Sweden). SARS-CoV-2 proteins were coupled to CM5 sensor chips using the amine coupling kit following the manufacturer’s instructions using 0.4 M 1-ethyl-3-(3-dimethylaminopropyl)-carbodiimide (EDC)/0.1 M N-hydroxysuccinimide (NHS) in water followed by ethanolamine deactivation (Cytiva Life Sciences). SARS-CoV-2 proteins used for interaction studies were prepared in 10 mM sodium acetate buffer with indicated pH values and immobilized at the indicated reference units (RUs): SARS-CoV-2 ORF7a (pH 5, 2500 RUs) SARS-CoV-2 ORF8 (pH 4, 1500 RUs), SARS-CoV-2 Spike_Strep_ (pH 4, 8000 RUs), SARS-CoV-2 Spike_His_ (pH 4, 12000 RUs), SARS-CoV-2 RBD (pH 4.5, 1500 RUs), SARS-CoV-2 S1e (pH 4.5, 6000 RUs), SARS-CoV-2 S2 (pH 3.5, 5500 RUs). On each CM5 sensor chip, a reference surface was prepared where blank was immobilized with EDC/NHS followed by ethanolamine deactivation. The signal detected for the reference surface was used for correction of non-specific binding. To verify the functionality of the ORF8-coupled sensor chip a Strep-Tactin-HRP conjugate (IBA Lifescience, Germany) was used as a control. Recombinant hACE2 was used to verify the functionality of all other SARS-CoV-2 protein-coupled sensor chips. Interaction studies were performed by injecting increasing concentrations (0-1280 nM) of the analyte in HBS-EP buffer (0.01 M HEPES, pH 7.4, 0.15 M NaCl, 3 mM EDTA, 0.05% (v/v) surfactant P20) (Cytiva Life Sciences, Germany). Injection cycles started with 3 min association followed by 3.5 min dissociation with a flow rate of 30 µl/min. Afterwards, a regeneration step with 10 mM glycine pH 1.5 for 30 sec was used. Kinetic constants were calculated by nonlinear fitting (1:1 interaction model with mass transfer) to the association and dissociation curves, as this model is default (BIAevaluation version 3.0 software). Apparent equilibrium dissociation constants (K_D_ values) were then calculated as the ratio of the dissociation rate constant (k_d_) and the association rate constant (k_a_). Experiments were performed in quadruplicates.

### Binding competition studies using surface plasmon resonance

For competition experiments, SARS-CoV-2 Spike_Strep_ was immobilized at 9000 RUs and 0-2560 nM LL-37 was injected followed by a subsequent 10 nM injection of hACE2 (“coinject” mode). Competition experiments with SARS-CoV-2 RBD, and SARS-CoV-2 S1e were performed by injecting 0-2560 nM LL-37 followed by an immediate injection of 640 nM hACE2 (“coinject” mode). Competition effects were detected as the percentage loss of signal compared to prior buffer injection. The absolute IC_50_ value was calculated using linear tendency of the curves.

### Negative stain electron microscopy

The structure of Spike and ORF8 alone and together with LL-37 or the control peptide were visualized by negative staining electron microscopy as described previously (Bober et al., 2010). Briefly, samples (usual concentrations 10 to 20 nmol/L) were incubated on carbon-coated grids for 1 min, washed and then stained with 0.75% uranyl formate for 1 min. Grids were rendered hydrophilic by glow discharge at low pressure in air. Specimens were examined in a Philips/FEI CM 100 TWIN transmission electron microscope operated at 60 kV accelerating voltage. Images were recorded with a side-mounted Olympus Veleta camera with a resolution of 2048×2048 pixels (2k×2K) and the ITEM acquisitions software. For identification of the viral proteins, strep-tagged Spike and ORF8 were detected with a gold-labeled anti-Strep antibody. For visualization of binding, LL-37 (or control peptide) was directly conjugated with colloidal gold.

## Results

### Recombinant expression of SARS-CoV-2 proteins

To study the interaction of LL-37 with SARS-CoV-2 we used recombinantly expressed Spike with either a strep or a histidine tag (Monomer Spike_Strep_:139 kDa, Monomer Spike_His_: 136 kDa), S1e (83 kDa), RBD (26 kDa), S2 (65 kDa), the accessory protein ORF8 (Monomer: 16 kDa) and the accessory protein ORF7a (32 kDa) (**Figure 1A**). Recombinant hACE2 was used as a positive control. The integrity and purity of the proteins were checked by SDS-PAGE following Coomassie staining (**Figure 1B**). Proteins bands of the expected size could be detected.

### Interaction studies using surface plasmon resonance

**Figure 2:**
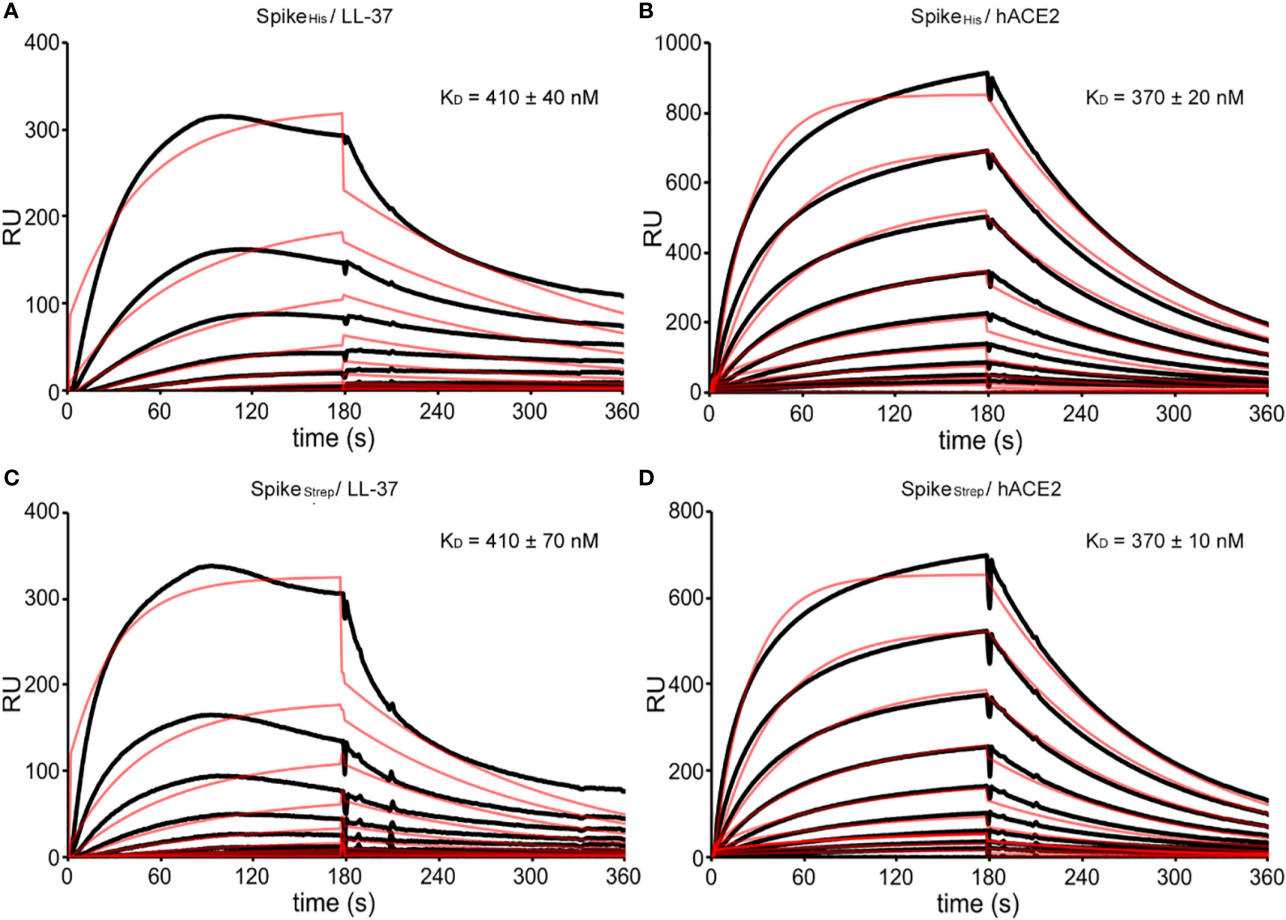
SPR sensorgrams with fitting curves showing the binding kinetics of Spike to LL-37 and hACE2, respectively. **(A)** Binding of LL-37 at 0-1280 nM to Spike_His_ (12000 RUs immobilized) and calculated equilibrium dissociation constant (K_D_). **(B)** Binding of hACE2 at 0-1280 nM to Spike_His_ (12000 RUs immobilized) and calculated K_D_. **(C)** Binding of LL-37 at 0-1280 nM to Spike_Strep_ (8000 RUs immobilized) and calculated K_D_. **(D)** Binding of hACE2 at 0-1280 nM to SARS-CoV-2 Spike_Strep_ (8000 RUs immobilized) and calculated K_D_. The fitting curves are shown as red lines. RUs are scaled.

To examine binding of LL-37 to Spike and to investigate the binding kinetics and interaction affinity of LL-37 with Spike by SPR, Spike_Strep_ and Spike_His_ were immobilized on a CM5 sensor chip (**Figure 2**). LL-37 showed consistent and specific binding to both Spike_His_ (**Figure 2a**, K_D_ = 410 nM) and the binding of Spike_His_ to hACE2 was measured as a positive control (**Figure 2b**). Similarly, LL-37 bound specifically to Spike_Strep_ (**Figure 2c**, K_D_ = 410 nM) and the binding of SpikeStrep to hACE2 was measured as a positive control (**Figure 2d**).

All sensorgrams of LL-37 showed a drop in the signal (RU) after approximately 100 sec. This phenomenon may be caused by oligomerization, which prevents further association of the monomeric analyte. It is known that LL-37 shows a concentration-dependent oligomerization and forms tetrameric channels essential for bacterial killing (Sancho-Vaello et al., 2017, 2020). While oligomerization reduces the availability of monomeric analyte, LL-37 can function in both monomeric and oligomeric forms, with larger complexes potentially enhancing binding to viral proteins such as the spike protein. This dynamic equilibrium may therefore contribute to its overall antiviral activity. Monomers and oligomers in solution can reach ratios of 4:6 (Xhindoli et al., 2013). The ratio changes depending on the buffer, and it is likely that the binding of LL-37 to a ligand will lead to a dynamic change in the ratio during the analysis according to the law of mass action. The dynamic change may lead to changes in the temporary interaction of LL-37 monomers and oligomers with the ligand. We assume that this is reflected by the slope of the association curves. We also conducted experiments with lower immobilization levels for Spike_Strep_, Spike_His_. Even at lower immobilization levels, the association curves did not show a plateau for optimal fitting of the curves. Furthermore, we used a default 1:1 interaction model with mass transfer to create the fitting curves. For an optimal fitting of the curves, a binding ratio between ligand and interaction partner must be specified. Since a different number of LL-37 molecules bind to Spike, it is not possible to specify an exact ratio. Nevertheless, we tested a different fitting model that resulted in K_D_ values of a similar order of magnitude. As a positive control binding of Spike to hACE2 was measured. Our calculated K_D_ value of 370 nM is higher than the K_D_ value of 15 nM found in literature (Wrapp et al., 2020). Repeated experiments that were adapted to the conditions used by Wrapp et al. (2020) with lower immobilization levels and changed buffer composition proved our measured binding affinities for Spike. A synthetic control peptide was used as negative control and did not show an interaction with the SARS-CoV-2 proteins (not shown).

**Figure 3:**
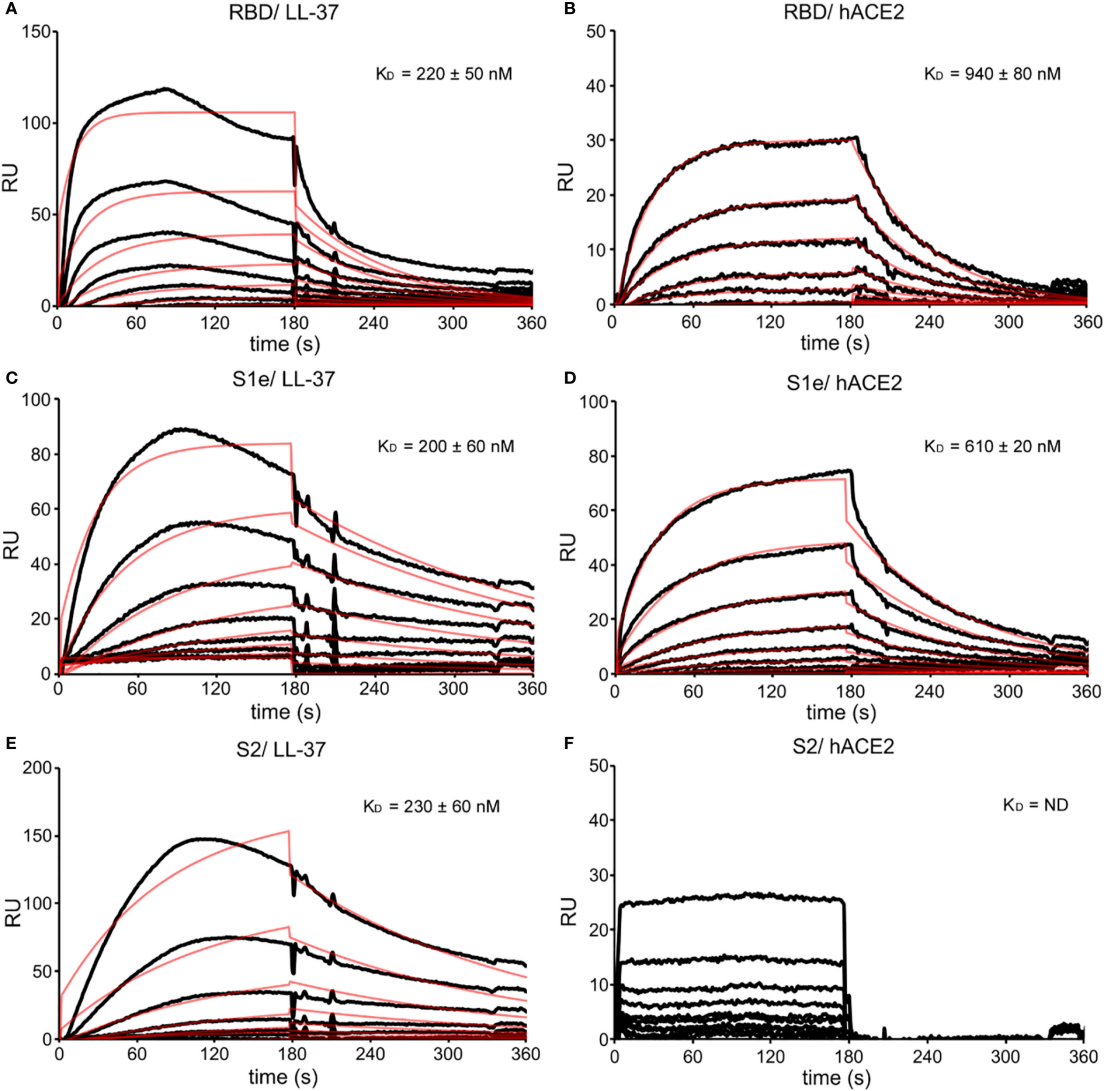
SPR sensorgrams with fitting curves showing the binding kinetics of RBD, S1e, and S2 to LL-37 and hACE2. **(A)** Binding of LL-37 at 0-1280 nM to RBD (1500 RUs immobilized) and calculated equilibrium dissociation constant (K_D_). **(B)** Binding of hACE2 at 0-1280 nM to RBD (1500 RUs immobilized) and calculated K_D_. **(C)** Binding of LL-37 at 0-1280 nM to S1e (6000 RUs immobilized) and calculated K_D_. **(D)** Binding of hACE2 at 0-1280 nM to S1e (6000 RUs immobilized) and calculated K_D_. **(E)** Binding of LL-37 at 0-1280 nM to S2 (5500 RUs immobilized) and calculated K_D_. **(F)** Binding of hACE2 at 0-1280 nM to S2 (5500 RUs immobilized) and calculated K_D_. The fitting curves are shown as red lines. RUs are scaled.

To define the binding site of LL-37 within the Spike, the recombinant proteins RBD, S1e, and S2 were immobilized on a CM5 sensor chip and the binding of LL-37 was analyzed (**Figure 3**). LL-37 bound to RBD (**Figure 3a**, K_D_ = 220 nM), and the binding of RBD to hACE2 was measured as a control (**Figure 3b**, KD = 940 nM). Similarly, LL-37 bound to S1e (**Figure 3c**, K_D_ = 200 nM),, with S1e-hACE2 interaction analyzed as a control (**Figure 3d**, K_D_ = 610 nM). LL-37 also bound to S2 (**Figure 3e**, K_D_ = 230 nM) with similar binding affinities. S2 showed no interaction with hACE2 (**Figure 3f**), demonstrating the specificity of the binding of RBD and S1e to hACE2, and of RBD, S1e, and S2 to LL‑37.

**Figure 4:**
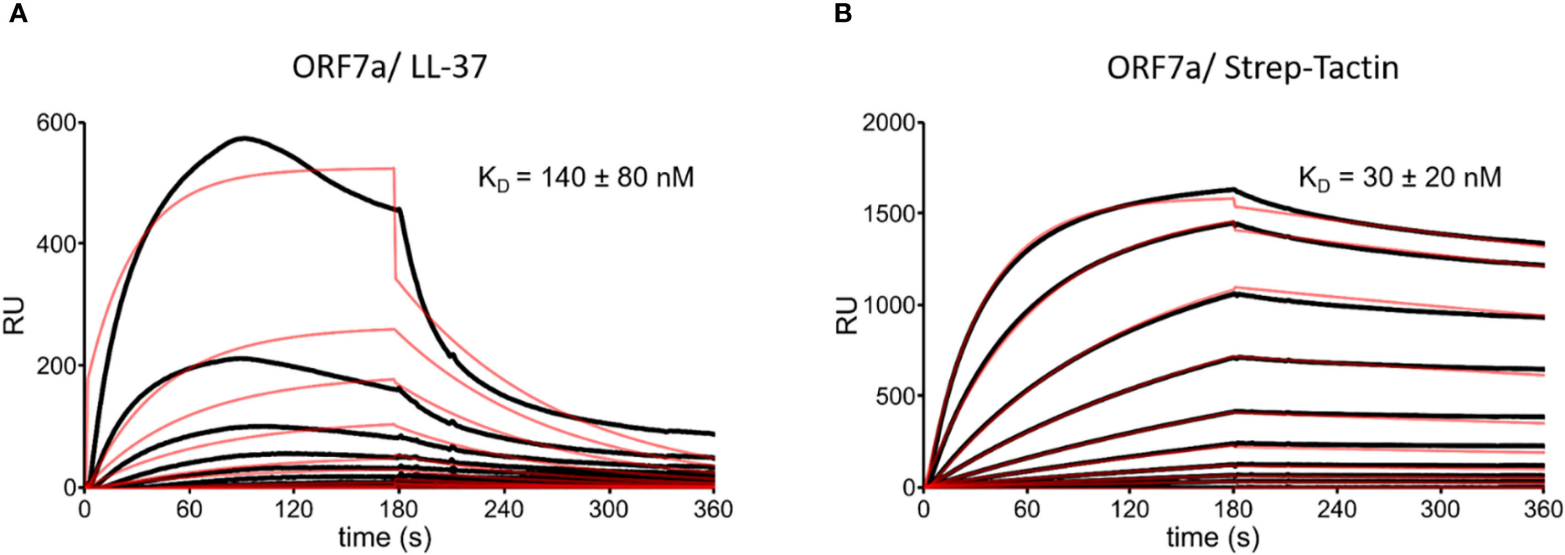
SPR sensorgrams with fitting curves showing the binding kinetics of ORF7a to LL-37 and Strep-Tactin-HRP conjugate. **(A)** Binding of LL-37 at 0-1280 nM to ORF7a (2500 RUs immobilized) and calculated equilibrium dissociation constant (K_D_). **(B)** Binding of Strep-Tactin-HRP conjugate at 0-1280 nM to ORF7a (2500 RUs immobilized) and calculated K_D_. The fitting curves are shown as red lines. RUs are scaled.

Since ORF7a boosts virulence by initiating autophagy and blocking autophagosome fusion with lysosomes (Hou et al., 2023), we analyzed the interaction of LL-37 and ORF7a (**Figure 4a**). With a measured binding with a K_D_ of 138 nM, the interaction of LL-37 and ORF7a was the most potent binding detected in our analysis. A Strep-Tactin-HRP conjugate was used to verify the functionality of the ORF7a coupled sensor chip (**Figure 4b**), as ORF7a carries a twin strep tag.

**Figure 5:**
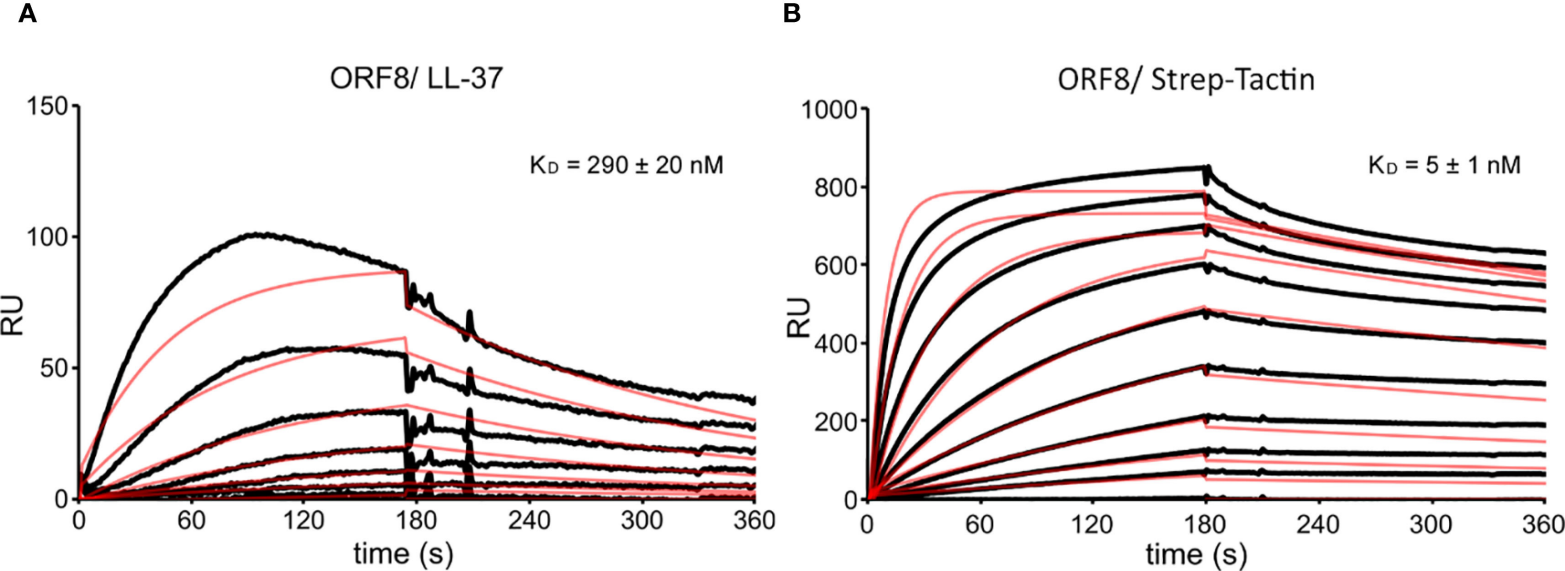
SPR sensorgrams with fitting curves showing the binding kinetics of ORF8 to LL-37 and Strep-Tactin-HRP conjugate. **(A)** Binding of LL-37 at 0-1280 nM to ORF8 (1500 RUs immobilized) and calculated equilibrium dissociation constant (K_D_). **(B)** Binding of Strep-Tactin-HRP conjugate at 0-1280 nM to ORF8 (1500 RUs immobilized) and calculated K_D_. The fitting curves are shown as red lines. RUs are scaled.

The accessory protein ORF8 is also regarded as a virulence factor (Hassan et al., 2021). Therefore, we also analyzed the interaction of LL-37 and ORF8 (**Figure 5**). Binding between LL-37 and ORF8 was detected with a K_D_ of 290 nM. A Strep-Tactin-HRP conjugate was used to verify the functionality of the ORF8 coupled sensor chip, as ORF8 carries a twin strep tag.

### Binding competition studies using surface plasmon resonance

**Figure 6:**
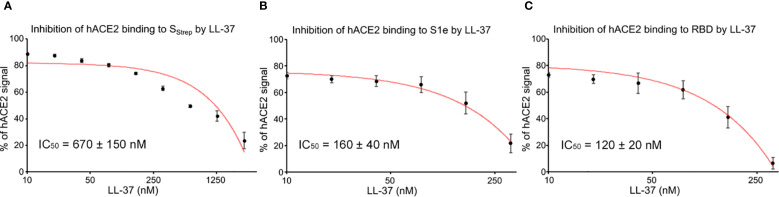
Analysis of the binding of hACE2 to Spike_Strep_, S1e, and RBD in the presence of LL-37 using SPR. **(A)** Dose-response curves for IC_50_ calculation for the LL-37 inhibition of Spike_Strep_ binding to hACE2. **(B)** Dose-response curves for IC_50_ calculation for the LL-37 inhibition of S1e binding to hACE2. **(C)** Dose-response curves for IC_50_ calculation for the LL-37 inhibition of RBD binding to hACE2. Average and standard deviations are shown in the dose-response curves. n = 3.

Competition studies were performed to investigate the binding of Spike, S1e, and RBD to hACE2 in the presence of LL-37. Therefore, sensor chips were immobilized with Spike_Strep_, RBD, and S1e, respectively, and hACE2 was injected with co-injection mode at a constant concentration directly after various concentrations of LL-37 (**Figure 6**). LL-37 IC_50_ values (LL-37 concentration, that blocks 50% of hACE2 binding to its ligand) for the binding of hACE2 to Spike, S1e, and RBD in the presence of LL-37 were calculated. LL-37 was able to reduce the binding of Spike_Strep_ (IC_50_ = 670 nM), S1e (IC_50_ = 160 nM), and RBD (IC_50_ = 120 nM) to hACE2. Our SPR interaction studies show binding of LL-37 to the S1e, S2, and RBD domains. The results of the SPR competition studies show a 6-fold higher IC_50_ value of LL-37 for Spike compared to RBD. More LL-37 molecules are required to block the binding of hACE2 to Spike, because LL-37 binds not only to RBD but also to other domains of Spike as shown by SPR. Based on the ratios of the K_D_ for RBD and Spike, six LL-37 molecules bind to Spike.

### Negative stain electron microscopy

**Figure 7:**
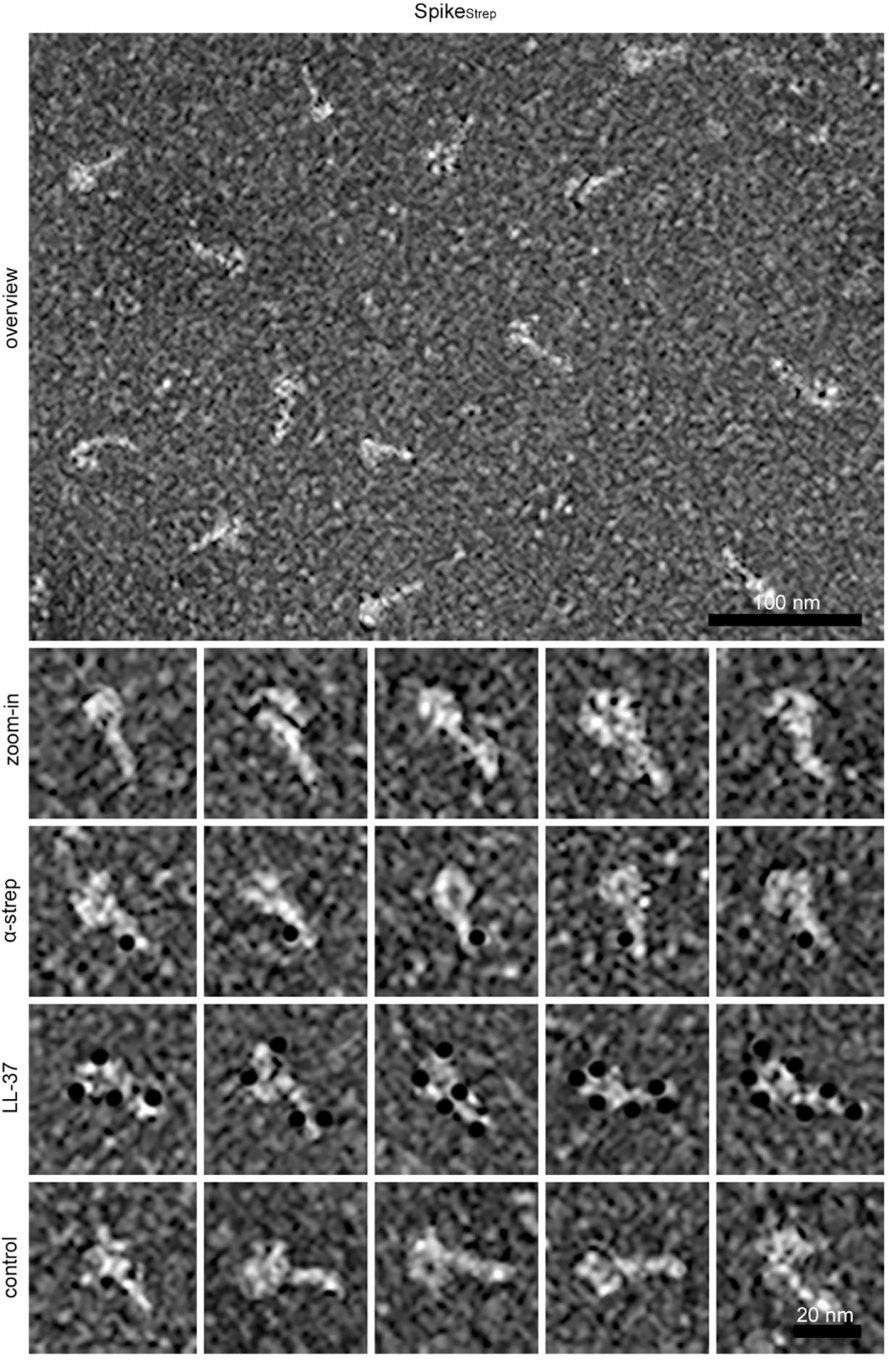
Negative stain electron microscopic images showing the binding of LL-37 to Spike. Overview and higher magnification (zoom in), immunolabeling of Spike_Strep_ with a gold-labeled anti-strep antibody (α-strep), and binding of gold-labeled LL-37 (LL-37) to Spike_Strep_. As control, a 29 amino acid long a-helical peptide was used (control). Black dots represent gold particles used for labeling the anti-strep antibody, LL-37, and a control peptide. The scale bar length of the overview is 100 nm and of the zoom-ins 20 nm.

To visualize binding of LL-37 to Spike we performed negative stain electron microscopy (**Figure 7**). The identity of Spike_Strep_ was demonstrated with a gold-labeled anti-strep antibody, which binds to the C-terminal strep-tag located in the S2 subunit. In the micrographs, Spike appears as a lollipop-shaped particle with a small tail corresponding to S2, where the gold-labeled anti-Strep antibody binds. LL-37 binds to the globular region (S1 subunit) as well as to the tail (S2 subunit), creating a halo-like structure around Spike with four up to a maximum of seven LL-37 molecules. LL-37, which was directly gold-labeled, was observed bound to both the globular head region (S1 subunit) and the tail region (S2 subunit), forming a halo-like structure around Spike with four to a maximum of seven LL-37 molecules. This agrees with the mean binding ratio of 6:1 determined in the SPR competition studies.

**Figure 8:**
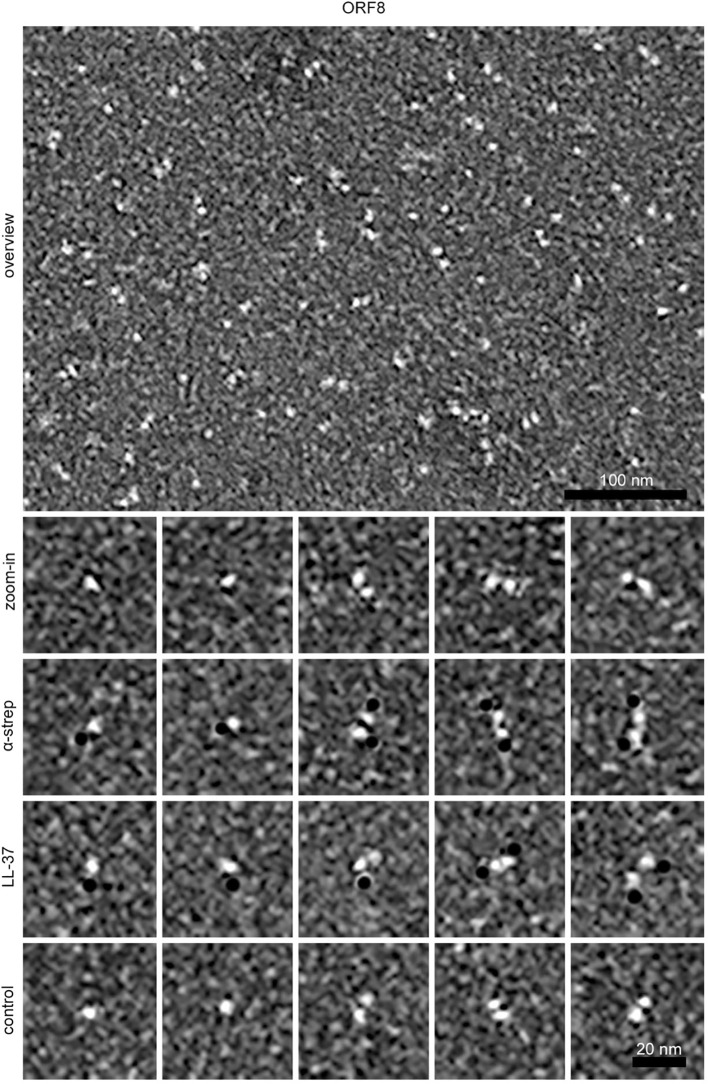
Negative stain electron microscopic images showing the binding of LL-37 to ORF8. Overview and higher magnification (zoom in), immunolabeling of ORF8 with a gold-labeled anti-strep antibody (α-strep), and binding of gold-labeled LL-37 (LL-37) to ORF8. As control, a 29 amino acid long a-helical peptide was used (control). Black dots represent gold particles used for labeling the anti-strep antibody, LL-37, and a control peptide. The scale bar length of the overview is 100 nm and of the zoom-ins 20 nm.

Binding of LL-37 to ORF8 was also investigated by negative stain electron microscopy (**Figure 8**). Strep-tagged ORF8 was identified using the gold-labeled anti-Strep antibody, appearing as small monomeric or dimeric globular molecules. Gold-labeled LL-37 was found bound to ORF8, with one LL-37 molecule associating with each monomer.

## Discussion

With the development of vaccines against SARS-CoV-2, the incidence of severe COVID-19 could be significantly reduced. Unfortunately, vaccination does not lead to complete protection against COVID-19, and severe courses are still possible. Nevertheless, vaccination is a critical tool in combating the pandemic. Due to the high rate of mutations of SARS-CoV-2, new virus variants frequently emerge that are less well recognized by the vaccines. Accordingly, vaccines must be continuously adapted to the new virus variants. Moreover, the rapid decrease of plasma antibody concentration after vaccination reduces the protection, so that additional therapeutic approaches may be required. ORF8 is thought to play an important role in evading the immune response of SARS-CoV-2 infection. However, there are currently no approaches to counteract the effects of ORF8, in part due to its low immunogenicity. Several studies describe the positive effect of vitamin D on the course of COVID-19 (AlSafar et al., 2021; Ricci et al., 2021; Sharun et al., 2021). Based on the fact that LL-37 is regulated by vitamin D, it was speculated that vitamin D could have a beneficial effect on the course of COVID-19 through increased formation of LL-37 (Crane-Godreau et al., 2020). Therefore, we investigated the interaction of LL-37 with Spike, ORF7a and ORF8. By surface plasmon resonance we demonstrated that LL-37 binds to different subdomains (RBD, S1e, S2) of Spike. Binding of LL-37 to Spike is not to a specific protein domain, but rather through widespread electrostatic or hydrophobic interactions or its amphipathicity. This assumption is supported by the fact that the antiviral effectivity of LL-37 against influenza infection *in vitro* or *in vivo* was not altered by replacing L-amino acids with D-amino acids, indicating that its action is not dependent on specific receptor interactions (Barlow et al., 2011). Interestingly, peptide binding to non-RBD regions can also reduce the attachment of Spike to hACE2 (Qiao and Olvera de la Cruz, 2020). We were also able to show that interaction of LL-37 with Spike is strong enough to inhibit the attachment of Spike to its cellular receptor hACE2 at nanomolar concentrations. Using electron microscopy, we could visualize binding of LL-37 to Spike. We detected up to seven LL-37 molecules simultaneously binding to Spike across the entire molecular surface of Spike, creating a halo-like structure. Considering that the binding of LL-37 can prevent the interaction of Spike and hACE2, the binding of up to seven LL-37 molecules makes an interaction of Spike with additional ligands unlikely. These results differ from those reported for human β-defensin 2 (hBD-2), which specifically binds to the Spike RBD and blocks its interaction with ACE2 (Zhang et al., 2022). In contrast, our data suggest that LL-37 does not act through a single RBD-specific interaction, but rather through widespread binding across multiple regions of Spike. Moreover, in silico studies have proposed that LL-37 may also bind directly to ACE2 (Li et al., 2021), raising the possibility that its antiviral activity involves interference with both Spike and its receptor. Together, these observations underline the versatility of LL-37 compared to other host defense peptides. Moreover, since LL-37 binds to Spike over the entire molecular surface, it may be that LL-37 binds not only to the Wuhan variant tested in this study, but also recognizes other virus variants. The shielding of viral surface proteins could be a general molecular mechanism by which antimicrobial peptides, as part of the innate immune system, exert antiviral effects. Meanwhile, the prevention of cell infection with SARS-CoV-2 by LL-37 was demonstrated with a pseudovirus assay and the therapeutic and preventive *in vivo* effectiveness was shown in mice (Wang et al., 2021). Our results reinforce the hypothesis that LL-37 may exert great beneficial effects in counteracting SARS-CoV-2 infections. An indirect therapeutic application of LL-37 would be conceivable by administration of vitamin D and the following induction of LL-37, but also directly by local administration, for example through inhalation. *In vitro* experiments predicted cytotoxicity of LL-37 (Oren et al., 1999; Säll et al., 2013). However, in plasma, up to 90% of LL-37 is bound to apolipoprotein A-I, which attenuates its cytotoxic effects *in vivo* (Svensson et al., 2017). Concerning a direct therapeutic application of antimicrobial peptides like LL-37, it is noteworthy that their pharmaceutical use has received approval for clinical trials (Koo and Seo, 2019). This could help to accelerate the use of LL-37 in the treatment of COVID-19.

In addition to LL-37’s demonstrated ability to bind and inhibit the SARS-CoV-2 Spike protein, we also explored its interaction with other viral proteins that contribute to the pathogenicity of the virus. Specifically, we investigated LL-37’s binding to ORF7a, another crucial factor in the virulence of SARS-CoV-2. ORF7a enhances viral replication by manipulating autophagy, initiating the process while inhibiting the fusion of autophagosomes with lysosomes through the degradation of SNAP29. This allows the virus to evade degradation within the host cell and sustain viral replication (Hou et al., 2023). Interestingly, LL-37, which is internalized by macrophages through a P2X7 receptor-mediated process and traffics to endosomes and lysosomes (Tang et al., 2015), could play a critical role in counteracting the effects of ORF7a. By binding to ORF7a, LL-37 may restore normal autophagic processes, preventing the virus from hijacking cellular machinery and enhancing viral degradation within lysosomes. Thus, LL-37’s ability to interfere with both Spike-mediated viral entry and ORF7a’s manipulation of autophagy highlights its potential as a multifaceted therapeutic against SARS-CoV-2. Combined with the fact that LL-37 can be induced by vitamin D, this positions LL-37 as a promising candidate for both direct therapeutic applications and supportive treatments aimed at mitigating severe COVID-19 outcomes.

Currently, there are no therapeutic approaches available to counteract ORF8. Multiple functions of ORF8 have been proposed, including disruption of antigen presentation via downregulation of MHC I expression and inhibition of interferon I signaling (Li et al., 2020; Zhang et al., 2021). Possibly, ORF8 is crucial for the high infectivity of SARS-CoV-2. Secretion of pro-inflammatory factors induced by ORF8 is mediated through activation of the IL-17 signaling pathway, which might contribute to a higher risk of thrombosis and acute respiratory distress syndrome (Pacha et al., 2020; Raucci et al., 2020; Lin et al., 2021). Furthermore, a prospective observational cohort study revealed that a deletion of ORF8 led to lower concentrations of pro-inflammatory cytokines, chemokines, and growth factors that are strongly associated with severe COVID-19 (Young et al., 2020; Kohyama et al., 2022). ORF8 is a potential candidate for vaccine development. Due to the low immunogenicity of ORF8 it is difficult to produce effective vaccines. Here, we demonstrate that LL-37 binds to ORF8 with similar binding affinities as those detected for binding of LL-37 to RBD, S1e and S2. The affinities were strong enough to inhibit binding of Spike to ACE2. The binding of two LL-37 molecules on the two opposite sides of the ORF8 dimer could result in the protein being shielded from further binding partners, as in Spike. Binding of LL-37 to ORF8 may mitigate its effects leading to a lower infectivity of SARS-CoV-2 and a less serious course of COVID-19.

LL-37 has not only antimicrobial and antiviral activities but also immunomodulatory properties. Influenza-infected mice showed reduction of granulocyte-macrophage colony-stimulating factor (GM-CSF), RANTES, and interleukin 1ß in bronchoalveolar lavage fluid after LL-37 treatment compared to the infected control group (Barlow et al., 2011). Furthermore, it was reported that higher serum levels of LL-37 correlate with reduced IL-17 expression in tonsils (Elenius et al., 2017). In the context of COVID-19, an excessive inflammatory response, also called hyperinflammatory syndrome, can occur. It was shown that an early administration of inhaled glucocorticoids reduces the likelihood of needing urgent medical care and decreases recovery time after early COVID-19 (Ramakrishnan et al., 2021). As severe COVID-19 patients show high levels of cytokines including IL-6, GM-CSF, RANTES, and IL-8, the immunomodulatory properties of LL-37 may have protective effects and improve patient survival rates (Huang et al., 2020; Tang et al., 2020). Nevertheless, LL-37 is closely linked to neutrophil extracellular trap (NET) formation, or NETosis, a process with both protective and potentially harmful consequences in COVID-19. LL-37 has been shown to promote NETosis (Radic and Muller, 2022), and excessive NET formation has been associated with tissue damage and immunothrombosis in severe disease (Anzic et al., 2025). In addition, it should also be considered that LL-37 has been reported to influence coagulation pathways. Elevated plasma levels in COVID-19 patients have been associated with markers of hypercoagulation and with enhanced activity of coagulation factors such as thrombin and factor Xa (Duan et al., 2022).While these findings highlight the need for careful evaluation of potential side effects, they do not diminish the overall therapeutic promise of LL-37, whose combined antiviral, antimicrobial, and immunomodulatory properties may still provide significant benefit.

## Data Availability

The original contributions presented in the study are included in the article/**Supplementary Material**. Further inquiries can be directed to the corresponding author.
