# Supplementary material for: Vitamin D-inducible antimicrobial peptide LL-37 binds SARS-CoV-2 Spike and accessory proteins ORF7a and ORF8
Source: Front Cell Infect Microbiol. 2025 Sep 23;15:1671738. doi: 10.3389/fcimb.2025.1671738 (PMC12500691; doi:10.3389/fcimb.2025.1671738)
Supplement: Supplementary file 1 [file Table1.docx]

#

# Vitamin D-inducible Antimicrobial Peptide LL‑37 binds SARS‑CoV‑2 Spike and Open Reading Frame Proteins 7a and 8

Annika Roth^1,†^, Steffen Lütke^2,3,†^ , Matthias Mörgelin^4^, Denise Meinberger^1^, Gabriele Hermes^1^, Gerhard Sengle^2,3,5,6,7^, Manuel Koch^2,6,8^, Marco Drexelius^9^, Jan Gebauer^9^, Ines Neundorf^9^, Dzemal Elezagic ^1*^,Mats Paulsson^2,5,6^, Thomas Streichert^1^_,_ Andreas R. Klatt^1^

Supplementary Table S1: Primer sequences and restriction enzymes. Restriction sites are marked with capital letters.

| Protein | Primer sequence | Restriction enzyme |
| --- | --- | --- |
| Spike protein fw | ccgaCGTCTCaagcttctgggcaacgtgctggttattg | Esp3I (HindIII) |
| Spike protein rev | cggtGGATCCttgttcatacttccccaactc | BamHI |
| RBD fw | ccacGCTAGCaatataaccaatttgtgtccg | NheI |
| RBD rev | ggtGGATCCttagaccgtggccggtgcgtgcaaaag | BamHI |
| Extended S1 subunit fw | accaTCTAGAcagtgcgtgaatcttaccac | XbaI |
| Extended S1 subunit rev | cgttagatcttaaacactgatagtaaagttgg | BglII |
| S2 subunit fw | ccagCGTCTCtctagcaagcgtcgccagtcaatcta | Esp3I (NheI) |
| S2 subunit rev | cggtggatccttgttcatacttccccaactc | BamHI |
| ORF7a fw | accaGCTAGCgagctttatcactaccaagagtg | NheI |
| ORF7a rev | gtgtggatcctcaaagttcttgaacttcctc | BamHI |
| ORF8 fw | accaGCTAGCgcatttcaccaagaatgtag | NheI |
| ORF8 rev | ggtGGATCCttagatgaaatctaaaacaac | BamHI |
| hACE2 fw | ccacGCTAGCAaccattgaggaacaggccaag | NheI |
| hACE2 rev | ctgtgcggccgcactccagtcggtactccatcc | NotI |
